# High-risk human-caused pathogen exposure events from 1975-2016

**DOI:** 10.12688/f1000research.55114.1

**Published:** 2021-08-04

**Authors:** David Manheim, Gregory Lewis

**Affiliations:** 1 1DaySooner, Delaware, USA; 2 Future of Humanity Institute, University of Oxford, Oxford, UK

**Keywords:** Laboratory Acquired Infection, Biosafety, Biosecurity, Laboratory Safety, Laboratory Accidents, Biological Warfare, Global Catastrophic Biological Risk

## Abstract

Biological agents and infectious pathogens have the potential to cause very significant harm, as the natural occurrence of disease and pandemics makes clear. As a way to better understand the risk of Global Catastrophic Biological Risks due to human activities, rather than natural sources, this paper reports on a dataset of 71 incidents involving either accidental or purposeful exposure to, or infection by, a highly infectious pathogenic agent.

There has been significant effort put into both reducing the risk of purposeful spread of biological weapons, and biosafety intended to prevent the exposure to, or release of, dangerous pathogens in the course of research. Despite these efforts, there are incidents of various types that could potentially be controlled or eliminated by different lab and/or bioweapon research choices and safety procedures.

The dataset of events presented here was compiled during a project conducted in 2019 to better understand biological risks from anthropic sources.

The events which are listed are unrelated to clinical treatment of naturally occurring outbreaks, and are instead entirely the result of human decisions and mistakes. While the events cover a wide range of cases, the criteria used covers a variety of events previously scattered across academic, policy, and other unpublished or not generally available sources.

## Listing events relevant to human-caused biocatastrophes

While there are certainly large-scale risks from natural pathogens ^1^, there is a likely far larger risk that emerges from various intentional human uses of pathogens ^2^, especially pathogens that have bioweapon or pandemic potential. This paper attempts to provide a list of all recent publicly known events that occurred due to research into or use of pathogens that could cause widespread damage. This includes any which are likely covered by the US Select Agents program, as well as other pathogens known to be capable of pandemic spread. This section begins with a discussion of inclusion criteria, then reviews issues with incompleteness of records, and finally discusses related prior literature, before presenting the list of events.

### Inclusion criteria

The time period and other inclusion criteria are necessarily somewhat arbitrary, but these events are thought to best illustrate the current risks of what researchers have referred to as human-caused Global Catastrophic Biological Risks (GCBRs). Because the events differ greatly, the list is split into separate categories with slightly different criteria. For biological weapons and related incidents, the list is restricted to events that occurred after the passage of the 1975 Biological Weapons Convention (BWC). This time period excludes both intentional spread of disease throughout human history, as well as somewhat recent events more closely associated with risks considered here, though the latter will be briefly mentioned.

For laboratory accidents, the time frame is identical, both for consistency, and because the modern era of lab safety began with significant changes after the first Asilomar conference in 1973 that identified a variety of risks and important needed changes to safety protocol. While the Asilomar conference was specific to recombinant DNA research, it led to changed practices more widely. Among other critical biosafety advances, this included the explicit identification of the dangers of ”mouth pipetting” ^3^, and following this, the 1975 conference focused in part on laboratory safety.

To focus on high-risk events, the inclusion criteria used is that the event involved a pathogen (either wild-type or a enhanced or weaponized variant) that is usable as a bioweapon, or that requires similar safeguards. Most events identified, therefore, involve agents that Pal *et al.* include as having a “probability to be used as bio-weapons” ^4^. This excludes, for example, laboratory HIV infections due to mishandled specimens ^5^, the seemingly frequent incidents of laboratory-acquired Brucellosis ^6^, and many other such events reported in the literature which pose important risks, but are far more limited in their impact than the events considered in this paper.

In listing events, we note that risks from potentially weaponisable pathogens can arise from a) their use in a biological attack, b) accidents involving their use within a biological weapons program, c) accidents involving their use within research for research purposes. As discussed below, laboratory acquired infections (LAIs), i.e. accidental exposure and infection during research, is the most common of the publicly known type of event. At the same time given secrecy and incomplete reporting, while we have moderate confidence that they are the most common, it seems far less likely that they are the most worrisome.

### Methods of data collection

A non-systematic search for laboratory accidents, use of biological weapons, and other related anthropic sources of risk was conducted. The search collated extant reviews and compilations from both academic literature, grey literature, and press articles, as well as finding additional events from each source. In each case, where possible the original sources and lists of additional events were investigated. The sources and source types are discussed in more detail below.

### Incompleteness of records

It is near-certain that there are events involving biological weapons that are, and will remain, unknown. Even research and development of biological weapons is forbidden by the BWC, so that violations of this convention, and any attendant accidents, would be expected to be kept secret. Furthermore, any deliberate release of a biological weapon would constitute a flagrant violation of international law, and a moral outrage. Such events are likely war crimes or crimes against humanity, and excepting rare cases like the truth and reconciliation commission in South Africa, it is unlikely that perpetrators will document their crimes or provide evidence to the public.

Where the BWC is not relevant, in the case of accidents in academic or permitted defensive research, there are still clear incentives at the individual actor, research, and national levels not to report. Individual workers are likely to be blamed for accidents, and may be hesitant to report them even when all proper precautions were taken. Laboratories and research projects where accidents occur are likely to be reprimanded, lose funding, or worse. The programs and governments that sponsor such research also have reputational risks when news of such incidents leak. In addition, Kahn suggests that the claim that secrecy is helpful makes failure to report accidents easy ^7^. For all of these reasons, it is unsurprising that the vast majority of known incidents are known only in retrospect, often after other parties reveal them - and this dynamic is made clear by the sources of reports included in this paper.

Despite all of these incentives, incidents become known through a variety of channels. This paper is an attempt to capture all such currently publicly known events, based on a review of news stories, books, gray literature, and academic journal articles on high-risk pathogen accidents and purposeful exposures. The broad scope is necessary because of the fragmentary reporting of such events.

Searches restricted to academic literature are notoriously incomplete. For example, of the 71 events identified in this paper, Su *et al.*’s 2019 review of Pubmed papers found only three reports - one Brucellosis infection, the 2009 death of Malcolm Casadaban due to Plague, and one case, reported by McCollum *et al.* but not included in other lists, of a laboratory worker that contracted Cowpox ^8, 9^. The lack of overlap seems to be a clear indication that most cases that occur are not reported in the medical or academic literature.

Similarly, incidents reported in the press are limited to those that are newsworthy in some sense. This list includes events that were uncovered via later investigative reporting or other means, but many events are reported properly to authorities but never noted publicly ^1^. The press sometimes covers these events when there is a death, or when there is some issue that points to negligence or irresponsible behaviour, as occurred at Boston University in 2005 where an infection was not properly reported ^10^.

This list, or any other compiled in a similar fashion, will be at best a generously underestimated lower bound on the historical rate of incidents in the United States. Further, given the paucity of non-US events, the list is plausible a far greater underestimation of the risk outside of the US. It is very likely that non-public records exist that will be uncovered in the future due to investigation, or will be declassified by governments based on public pressure. Not only should it be expected that the list will need to be revised in the future to account for new revelations and future events, but we should expect that some events will never be known to the public, perhaps including major accidents or intentional acts.

### Previous work and sources

There have been many prior lists of events, but while sources reviewed vary, none are complete. Even if reports were intended to be comprehensive, they become outdated as additional historical events become known, and as additional events occur. Along with mention of key sources, we will note issues with both academic sources and gray literature, as well as the limitations of news reporting on such events.

***Academic literature*.** In the academic literature, many accounts are case studies and descriptions rather than attempts to provide a catalogue of events, but these have been compiled in several review articles. Unfortunately, the focus differs markedly and while they cover lab accidents of various types, most focus on clinical accidents. (Healthcare workers are known to be at high risk of exposure to these pathogens when they occur ^11^, but these infections are due to necessary clinical work and so are unrelated to bioweapons or research, thus are excluded here.) They also are incomplete, at least in part because the source case studies rely on self-reporting.

Early work compiling accidental exposures includes Hanson *et al.*’s 1967 paper that attempted to catalogue laboratory acquired infections ^12^, which was updated by Pike to cover up until 1975 ^13^, though this almost entirely predates the time period being considered. Richardson also presented a summary of 109 LAIs that occured at the Centres for Disease Control (CDC) from 1947–1973 at the 16th Annual Biosafety Conference, though this seems not to have been published ^14^. More recently, Harding and Byers surveyed case report and other academic literature from 1979–2004, and Harding and Byers later extended the dataset to 2015 ^15^. Both of these lists did not fully split out infections between research, clinical, and diagnostic laboratories, but the earlier review noted that most reported bacterial infections occurred in clinical laboratories (84 vs. 471) while most reported viral infections occurred in research facilities (418 vs. 181). The later review similarly found that ”Sixty-seven percent of the viral LAIs occurred in research facilities”, while less than 10% of bacterial infections were in research laboratories.

Many or most of the laboratory-acquired infections reviewed in Harding and Byers were related to the accidental use of or exposure to infected animals, rather than intentional work with the pathogens, and many were low-consequence infections. While this makes many cases not relevant for the current review, the initial paper found 155 cases and one death from Hantaviruses, 143 cases of Brucella ^2^, 5 cases of Chikangunya (2 asymptomatic), 4 cases of Dengue, and 6 cases of SARS-CoV. The update to this work in 2017 by Harding and Byers ^15^ extended the dataset to 2015, and included another death and 108 additional clinical and subclinical infections from Hantavirus, 235 additional cases of Brucella, 3 additional cases of Dengue, and no new cases of Chikangunya or SARS-CoV. Importantly, it seems the overall direction in number of such infections is ambiguous. A related effort, assisted by Byers, is the Association for Biosafety and Security (ABSA)’s searchable LAI database ^16^, which has been updated at least once since the initial release in 2016, and is currently publicly accessible. This seems to be an ongoing project to compile events, though the list is restricted to ”peer-reviewed published LAIs” and based on this review, seems to be unfortunately far from complete. (Note that a single event was found in this database which was not listed elsewhere, a potential exposure to Anthrax in a BSL-4 laboratory due to a ripped suit ^17^. Incidentally, the event was sourced to a newpaper article rather than a peer reviewed source.)

***gray literature*.** Some more recent work is gray literature that has focused more directly on some classes of research-related risk discussed here. For example, the 2008 Environmental Impact Statement (EIS) for the National Bio and Agro-defense Facility (NBAF) opened by the US government in Manhattan, Kansas had an appendix that detailed biocontainment lapses and LaIs relevant to research ^18^. Given more recent revelations, this is still somewhat dated, but it contains an attempt at a comprehensive list of relevant events. A recent paper in the Bulletin of Atomic Scientists discussed human error events and associated risks, and the appendix included two event lists for more recent events based on Federal Select Agent Program (FSAP) reports obtained via a Freedom Of Information Act (FOIA) request by John Greenewald, Jr. These reports, by the US Department of Agriculture and Health and Human Services to the US Congress, cover the years 2003–2015 ^19^. These lists unfortunately do not clearly distinguish between research accidents and clinical or diagnostic accidents, though for this paper we have inferred which events fit the criteria outlined below. It also has insufficient information to know what events are being reported, including where the event occurred or when. Another sources is a set of publicly released FOIA requests by the Sunshine Project covering 2003–2006 ^20^, discussed in more detail below.

Each of these sources are incomplete, and overlap in some places but not others. Many of the events found by the Sunshine Project are missing from the FSAP reports covering that period. The Select Agent report also misses several events in the United States that were reported publicly elsewhere. One recent report listing lab accidents in research is Silver’s 2015 in-depth review of five cases of laboratory-acquired lethal infections by potential bioweapon pathogens, which also mentions several other events. Silver includes several events that were included in neither the Select Agents program reports, nor the Sunshine Project work, nor the 2008 EIS. At the same time, Silver’s list does not claim to be comprehensive, and excludes many events that are listed elsewhere ^21^.

***Intentional use*.** All of these reports exclude intentional use a biological warfare as unrelated to laboratories. While this makes sense for research considering only LAIs, it ignores other risks, such as the fact that some intentional uses were only possible because of research being done. Misappropriation of the Anthrax used in the 2001 attacks was possible because of access to samples in a research laboratory, and training and experience in cultivating anthrax in the course of doing research.

Even more directly implicated are deaths due to intentional use in warfare. Frischknecht provides a very useful and comprehensive review of the history of biological warfare. Unfortunately, this includes little about of post-WWII biological warfare, many examples of which have come to light after that publication in 2003 ^22^. The literature on these is small, and scattered, with many more allegations than known cases, but events from several recent books and news articles on these events are included.

## Events

Some events involve human deaths, others involve either active infections or seroconversions (i.e. post-hoc detection of antibodies that indicate an infection occurred,) and some involve spread to animals, or a failure to spread despite definite exposure. Not included in the event counts are suspected but nonconfirmed exposures during research, or clinical laboratory accidents that occur during the treatment of natural outbreaks or diagnostics of naturally occurring disease, though both are also discussed briefly.

In the list, it is critical to differentiate between purposeful deployment of bioweapons and accidental events, and both Table 1 and the below discussion does so. It is also useful to further distinguish different classes of events. In addition to malicious intentional use, there is a range of purely defensive research - though this does not preclude risks due to accidents or misuse. Purposeful usage includes both misuse by rogue actors, testing of biological weapons, and purposeful deployment by state actors. Accidental exposures include both straightforward research lab accidents, exposure due to incomplete safety precautions, and accidental exposure due to confusion of dangerous pathogens with less-dangerous or inert samples.

**Table 1.** High risk human-caused pathogen exposure events from 1975–2016.

Year	Location	Pathogen	Program Type	Event Type	Spread
1978 ^*A*^	Plum Island, NY (US)	Foot-and-Mouth Disease	Biodefense	Accident	2 Steers ^*o*^
1977-78 ^*D*^	Global	H1N1 Influenza	Unclear	Accident	Millions infected ^23^
1978 ^*A*^	Plum Island, NY (US)	Foot-and-Mouth Disease	Biodefense	Accident	Many Cows ^*o*^
1978 ^*D*^	University of Birmingham Medical School (UK)	Smallpox	Biodefense	Accident	1 Killed
1979 ^*D*^	Sverdlovsk (Russia)	Anthrax	Bioweapon	Accident	100+ died ^25^
Late 1970s	South Africa ^+^	Cholera	Bioweapon	Intentional	Unclear
Late 1970s	Rhodesia	Anthrax (and others ^+^)	Bioweapon	Intentional	1,000s infected, 82+ died ^26^
1980 ^*A*^	Plum Island, NY (US)	Foot-and-Mouth Disease	Biodefense	Accident	9 Steer
1980s	Iraq	Various ^+^	Bioweapon	Human Testing	Unknown
1981 ^*A*^	Plum Island, NY (US)	Foot-and-Mouth Disease	Biodefense	Accident	4 Steer
1987 ^*A*^	Plum Island, NY (US)	Foot-and-Mouth Disease	Biodefense	Accident	1 Heifer
1990 ^*D*^	Koltsovo, Russia	Marburg	Biodefense	Accident	1 Infection ^27^
1993	Tokyo (Japan)	Anthrax	Non-State	Bioterrorism	Failure ^28^
1999 ^*D*^	Oblivskaya (Russia) ^+^	CCHF-like Virus	Bioweapon	(Alleged) Accident	69 Hospitalized, 6 dead ^29^
2000s Onward	North Korea	Various ^+^	Bioweapon	Human Testing	Unknown
2001 ^*D*^	United States Postal System (US)	Anthrax	Non-State	Bioterrorism	22 infected, 5 died
2002 ^*A*^	University of Texas, Houston (US)	Anthrax	Research	Accident	1 Infected
2002 ^*A*^	Fort Detrick, MD (US)	Anthrax	Research	Biodefense	No infections
2002 ^*A*^	Unclear (US)	West Nile Virus	Research	Accident	1 serroconversion, with mild symptoms
2003-2006	Unknown ^×^ (US)	Brucella	Research	Accident	1 Infected
2003 ^*D*^	Texas A&M (US)	Anthrax	Research	Accident	Missing Specimens
2003 ^*A*^	National Defense University, Taipei (Taiwan)	SARS-CoV	Research	Accident	BSL-4 facility, pathogen spread to BSL-2, 1 researcher infected
2003 ^*A*^	Singapore (US)	West Nile and Sars-CoV	Research	Accident	BSL-3 facility, 1 Student Infected
2003 ^*A*^	University of New Mexico (US)	Redacted ^*A*^, Anthrax ^*C*^	Research	Accident	1 Researcher received prophylactic treatment
2003 ^*A*^	Columbus, Ohio (US)	West Nile Virus	Research	Transit Accident	50 Federal Express workers exposed ^30^
2004 ^*A*^	National Institute of Virology, Beijing (China)	SARS-CoV	Research	Accident	BSL-2 laboratory, 2 researchers infected, spread to 6 ^*A*^ or 11 ^*D*^ in the community, and, one of the researcher’s mother, who was the ﬁrst identiﬁed case, died.
2003-2006 ^*B*^	Unknown ^×^ (US)	Brucella	Research	Accident	1 Infected
2003-4 ^*A*^	Infectious Disease Research, Inc., Seattle, WA (US)	Tuberculosis	Research	Accident	3 subclinical infections
2004	Plum Island, NY (US)	Foot-and-Mouth Disease	Research	Accident	2 Cattle
2004 ^*A, B, D*^	VECTOR (Russia)	Ebola	Biodefense	Needlestick	1 Killed
2004 ^*A*^	Children’s Hospital and Research Center, Oakland (US)	Anthrax	Research	Accident	5 Workers Exposed
2004 ^*A*^	Fort Detrick, MD (US)	Ebola	Biodefense	Needlestick	No infection
2004 ^*A*^	NIH, Bethesda, MD (US)	Various	Research	Infrastructure Accident	No Exposure
2004 ^*A*^	University of Illinois at Chicago (US)	Various	Research	Noncompliance	No Exposure
2004 ^*A, D*^	Boston University	Tularemia	Research	Accident	3 infections
2004 ^*A*^	UMDNJ (US)	Plague	Research	Missing Specimens	3 mice (intentional exposure.)
2004-5 ^*A, D*^	International	H2N2 Influenza	Other	Diagnostic Trial Test	Sent to 2,750 US Labs and 3,747 International Labs. No infections
2005 ^*A, C*^	Medical University of Ohio (US)	Valley Fever	Research	Aerosolization Accident	1 student exposed
2005 ^*A*^	UNC Chapel Hill (US)	Tuberculosis	Research	Biocontainment loss	no infections
2005 ^*A, C*^	University of Chicago (US)	Anthrax or Plague	Research	Accident	1 Possible infection
2005 ^*C*^	UC Berkeley (US)	Rocky Mountain Spotted Fever	Research	Accident	Exposure (extent unclear)
2005 ^*A*^	Lawrence Livermore National Laboratory to Palm Beach, FL (US)	Anthrax	Research	Transit Accident	2 workers exposed
2005-6 ^*A*^	University of Wisconsin Madison (US)	Ebola	Research	Misclassiﬁed	Treated as Biosafety Class (BSL-2)
2006 ^*A, C*^	Texas A&M (US)	Q-Fever	Research	Aerosolization Accident	3 Workers
2006 ^*A, C*^	Texas A&M (US)	Brucella	Research	Accident	1 Researcher
2006 ^*A*^	University of Texas Austin (US)	H3N2	Research	Accident	1 Researcher exposed
2006 ^*A*^	Texas A&M (US)	Q-Fever	Research	Missing Specimens	1 Mouse
2007 ^*A*^	Pirbright (UK)	Foot-and-Mouth Disease	Research	Accident	4 Farm Sites
2007 ^*A*^	University of Texas Houston Medical Center (US)	Anthrax	Research	Accident	4 Researchers exposed, prophylaxis refused
2007 ^*B*^	Unknown ^×^ (US)	Brucella	Research	Accident	1 Infected
2008 ^*B*^	Unknown ^×^ (US)	Brucella	Research	Accident	1 Cow Infected
2007 ^*A*^	University of Mississippi Medical Center	Anthrax	Research	Flask Break	1 Student Exposed
2008 ^*B*^	Unknown ^×^ (US)	Brucella	Research	Accident	1 Infected
2009 ^*D*^	Hamburg (Germany)	Ebola	Research	Needlestick	1 Researcher Exposed, Innoculated ^31^
2009 ^*D*^	University of Chicago (US)	Plague	Research	Accident	1 Killed ^21^
2009 ^*B*^	Unknown ^×^ (US)	Tularemia	Research	Accident	1 Infected
2010 ^*B*^	Unknown ^×^ (US)	Brucella	Research	Accident	1 Infected
2010 ^*B*^	Unknown ^×^ (US)	Brucella	Research	Accident	1 Infected
2010 ^*B*^	Unknown ^×^ (US)	Classical Swine Fever	Research	Accident	2 Animals Infected
2010 ^*D*^	Unknown (US)	Cowpox	Research	Accident	1 Infected ^8, 9^
2011 ^*D*^	Australia	Dengue	Research	Accident	1 Infected ^32^
2011	University of Chicago (US)	Anthrax	Research	Accident	
2012 ^*B*^	Unknown ^×^ (US)	Q-Fever	Research	Accident	1 Serroconversion
2012 ^*B*^	Unknown (Separate from Above) ^*x*^ (US)	Q-Fever	Research	Accident	1 Serroconversion
2013 ^*B*^	Unknown ^×^ (US)	Melioidosis	Research	Accident	1 Infected
2013 ^*B*^	Unknown ^×^ (US)	Brucella	Research	Accident	1 Infected
2014 ^*B*^	Unknown Veterinary Medical Teaching Hospital ^×^ (US)	Q-Fever	Research	Accident	2 Infected
2014	South Korea	Dengue	Research	Accident	1 Infected ^33^
2015 ^*B*^	Unknown University Research Lab ^×^ (US)	Q-Fever	Research	Accident	2 Serroconversions
2015 ^*B*^	Unknown Government Research Lab ^×^ (US)	Brucella	Research	Accident	1 Serroconversion
2016 ^*D*^	National Center for Foreign Animal Disease in Winnipeg (Canada)	Anthrax	Research	Accident	17

Sources: **A** National Bio-and Agro-Defense Facility's Environmental Impact Statement ^18^ **B** FSAP reports to Congress ^19^ **C** Reports uncovered by the Sunshine Project ^20^ **D** Individual reports and news articles ^o^ Many agricultural infections that infect animals outside of laboratories are not fully traced, but all livestock potentially exposed or infected are slaughtered. In such cases, the exact extent is unknown. ^+^ These are credible allegations, albeit with various degrees of support from published epidemiological research or documentation of the events. ^×^Discussed in DHS Select Agent reports, further details are unavailable.

As an introduction to the list of events, it is worth noting that even for events that are known and documented, there can be significant uncertainty. One useful example is the 1977 influenza epidemic, discussed by Rozo and Gronvall ^23^. There is now near-certainty that the influenza virus was introduced by human action, but the details of how this occurred are unclear. Reasonable theories include purposeful deployment, accidental release from bioweapons or related research, or accidental infection due to a live vaccine ^24^. Furthermore, details of the escape may not be known even to those involved. If the pathogen escaped from lab, the lab may not have been aware, and the connection to the live vaccine trials were made only decades later. In other cases, including events that are included in the list but not detailed in FSAP reports to the US Congress, the details of the events are potentially known and/or documented, but any known details are not available publicly.

### Event list

Table 1 summarizes the events that qualify given the criteria listed above. The various classes of programs and events, along with illustrative examples from the list, are detailed later in the paper. Note that while details of programs are typically unavailable. Because of this, accidents in locations known to be working on state bioweapons programs are listed as such, while accidents in laboratories and military locations openly working on bioweapons defense as such. Any other research not conducted as state biodefense work, or where details are unknown, is listed as (academic) research.

As noted in the notes Table 1, the primary sources for these incidents include the National Bioand Agro-Defense Facility’s Environmental Impact Statement ^18^, FSAP reports to Congress ^19^, reports uncovered by the Sunshine Project ^20^, and individual reports and news articles which are individually cited in the table.

## Discussion

The sources for the above events are both varied, and incomplete. Despite limitations, however, the events and the details that are known do allow a limited degree of insight into the historical risks. Given that, we review some of the events and make observations for each class of event. This review starts with intentional use, then accidents in bioweapons programs, and finally research laboratories. For each, we discuss the likely missing data, overall trends, and the various changes made to reduce risk, and some implications of the risk that remains.

### Intentional use

Intentional use of bioweapons is rare, and there are few recorded cases since the passage of the BWC, but the extent of devastation possible from such use makes them an important part of the overall risk. Several known purposeful uses of bioweapons, and allegations of such use exist ^3^, and these illustrate different sub-categories of risk; (1) intentional military use of biological weapons, including the alleged uses of various biological weapons by Rhodesia in the late 1970s, and the alleged use of Cholera by the South African military, (2) intentional testing of biological weapons on humans, as alleged in the Iraqi and North Korean bioweapons programs, and (3) misuse by a rogue and non-state actors, as in the 2001 Anthrax attacks in the United States, or the failed anthrax attacks by Aum Shunrikyo.

The clear trend away from large states pursuing biological weapons after the passage of the BWC is the subject of an extensive literature. Given that a key purported factor is norms, it is worth noting several notable events prior to the passage of the BWC, but well after the widespread recognition of the unacceptability of intentional or negligent exposure of civilians to bioweapons. These include the US government’s Tuskegee and Guatemalan syphilis experiments that ended by the early 1970s, and the various biological weapons programs during World War II.

### Intentional state bioweapon use

The first alleged intentional deployment of biological weapons after the passage of the BWC was the use of various biological weapons by the Rhodesian government ^26, 34^. This is perhaps best thought of as a series of events, the largest of which was the use of anthrax during the Rhodesian insurgency in 1978–9. While there is some dispute about the causes of this outbreak, recent analyses of the outbreak pattern strongly imply that it was not of natural origin ^26, 35^. In addition, Cross details the extensive use of various chemical and biological weapons during the Rhodesian wars by the government. Specifically, Cholera was intentionally released at least twice into rivers in order to infect black South African villages, and Anthrax was released at least once ^36^.

Closely related to, and partially allowing the Rhodesian incidents, South Africa developed and stockpiled a number of chemical and biological weapons. There have been at least some allegations that South Africa used the biological weapons they developed, but if any such use occurred, it was not documented by the extensive later investigations ^36^.

These types of intentional events are worrying, as states undergoing nationally existential crises, as Rhodesia did, could make similar decisions now or in the future ^37^. Thankfully, the rate of such wars and insurgencies has been declining ^38, 39^.

In addition to intentional attacks using developed biological weapons, it seems that various biological weapons programs have tested weapons on human subjects. Included in this are allegations that there was an Iraqi program that involved human testing of Anthrax in the 1980s ^40^. Similarly, there have been allegations that North Korea tested bioweapons on prisoners ^41^. This class of testing is itself a crime against humanity, and can pose further risks if the pathogen spreads. However, no such testing has been proven or more clearly documented as having occurred in any of these cases.

### Bioterrorism

Bioterrorism and misuse by rogue actors is an oft-mentioned concern, albeit with few examples of even attempted attacks ^4^ An earlier and more successful event was the 1984 Rajneeshee attack, a food-poisoning attack with Salmonella that led to 751 infections and 45 hospitalizations, but no fatalities. It is noteworthy, but does not involve a pathogen with biowarfare potential, per Pal *et al.* ^4^, and was intended as a method of short-term incapacitation rather than as a mass casualty attack ^42^. In addition to this attack, Carus lists a number of other attempts and actual attacks during the time period in question involving chemical agents and pathogens of minimal concern that do not qualify. The single exception was Aum Shinrikyo, which attempted both an anthrax attack and an attack using botulinum toxin (”botox”) against downtown Tokyo in the 1990s, which failed for a variety of reasons. Regarding Anthrax, Olson explains that “the cult may not have had the right agents or the right technologic facilities; they could have overcooked the bioagents or not known how to use them” ^28^.

The only bioterrorism attack involving a highly-dangerous pathogen that was successful involved the 2001 mailing of a series of envelopes with weaponized anthrax spores to a number of prominent US politicians. The attack was later determined to have been carried out by a US bioweapons researcher who stole an anthrax sample from the lab he was working in, then cultured it. One point that is perhaps notable for other reasons, despite the purely defensive nature of the research, the transition from defensive to offensive work is possible in at least some cases. The reaction to this event included numerous recommendations for better managing risks from Anthrax attacks, including Inglesby *et al.*’s summary ^43^, but these all focused on response and risk mitigation.

It is noteworthy that there has been little emphasis on mitigating risks by choosing not to train and employ scientists to cultivate samples of weaponized strains of anthrax or other bioweapon-capable organisms. This suggestion may seem naive, but there are a variety of reasons to think it is both viable, and would be effective. First, the difficulty of preventing insider threats is well documented ^45^. Second, not only is such cultivation complex (despite clear interest ^46, 47^), dangerous potential biological weapon pathogens have never been successfully used by terrorist groups. In fact, many such groups have attempted to develop and use pathogenic agents ^42^, so a large corpus of biodefence activity may pose a greater bioterrorist threat than the one it is trying to combat.

For example, the attempt by Aum Shinrikyo discussed above, along with their abortive attempts to acquire and culture other pathogens including Ebola, were only discovered in retrospect. Note that Aum Shinrikyo has access to far more money and resources than all but the most successful terrorist groups. Despite these resources, and the near-complete lack of effort devoted to stopping their work, their attempts and failure would still be unknown if not for their successful chemical weapon attack and subsequent investigation and prosecution. As noted earlier, the only recorded deaths from bioterrorism involved pathogens from a state bioweapons program, cultured by a researcher in that program.

### Accidental releases

There have been several classes of releases that could pose a threat of spread. First, there are accidents related to biological warfare program research. Second, there are accidental exposures during scientific research, including both accidental laboratory exposure, and exposures where the exact method of transmission is less clear. Third, there are exposures due to due to the incorrect labelling or distribution of a pathogen. Excluded from the list and counts in this paper, but discussed briefly, are infections of healthcare workers in the course of clinical work related to natural outbreaks.

***State bioweapon program accidents*.** Accidents involving (illegal) State-sponsored biowarfare research have occurred at least once, and near-certainly several other times since the enactment of the BWC in 1975. The first and only well documented case was the 1979 Sverdlovsk Anthrax Leak, in which anthrax being cultivated and processed for use in missiles was accidentally released due to a mis-communication about a filter which was removed for cleaning ^25^. A second event allegedly occurred in 1999, in Oblivskaya. At that time, Alexander Kouzminov notes that there was “an accidental release of a synthesized virus” that is similar to Crimean-Congo hemorrhagic fever, which caused a small outbreak, and infers that there was a cover-up ^29^.

An earlier event in the USSR, in what is now Kazakhstan, is the Aral smallpox incident. This occurred in 1971 and so predates the BWC, but despite being excluded form the list, it illustrates the potential for spread. Zelicoff and Bellomo ^48^ explains that the incident started due to a dispersal test of an enhanced strain of smallpox, which subsequently accidentally spread off of the island being used for the weapons test to a nearby ship, which docked, after which the infected individuals spread the infection further. This nearly led to a large-scale epidemic, and was stopped only by quarantining thousands of people for several weeks, incinerating several properties, halting all traffic in and out of a city, and launching a mass vaccination campaign including over 50,000 people.

Also prior to the time period discussed, it is worth mentioning the deaths of William Boyles, Joel Willard, and Albert Nickel. Per Shane ^49^, and per Treaster ^50^, all three were accidentally infected in the course of US bioweapons research at Fort Detrick in separate incidents in 1951, 1959, and 1964, respectively. Rusnak *et al.* ^51^ reviewed records from the US bioweapons program at USAMRIID from 1943 to 1969, starting before the routine use of modern biosafety equipment and the availability of vaccines, and found 423 infections during that period. While similar events are not known about various other countries’ Bioweapons programs, it would seem likely they occurred, despite the fact that such accidents are unknown, given that accidents in the course of pursuing bioweapons are unlikely to be publicised.

The lack of current events makes it tempting to conclude that the risk is now low, but the logic behind such a conclusion is faulty. The events mentioned here are limited to the US program and exposure of the public in the Russian program, and in each case, the events were kept classified, and the incidents were only revealed in retrospect. In the US, the fatalities were first uncovered due to congressional pressure on the US Army towards the end of the Vietnam war, and the much later paper by Rusnak *et al.* reviewing (still non-public) medical records revealed the hundreds of nonfatal cases. In Russia, the few events are known mostly due to defectors from the biological warfare program. But-for these reasons, it seems likely the US program, and the non-public infections and fatalities in Russia, would not have been uncovered - and it seems near-certain that accidents in other countries that pursued bioweapons occurred but remain undisclosed.

A more reasonable conclusion would be to consider this risk to be proportional to the (unknown) number and size of extant bioweapons programs. The risk of accidents in such laboratories is almost certainly lower than it once was, due to improved understanding of safety. How this compares to other laboratories is unknown.

### Research accidents

The largest category of known events are accidents in laboratories doing research on bioweapon relevant pathogens ^5^. While incidents that are known mostly occurred in the United States, it seems likely that this is due to a combination of the relatively large amount of research into these pathogens done there, and the ability for investigators to file FOIA and similar requests ^6^.

Most of the incidents have straightforward causes, such as accidental skin punctures from needles or otherwise, or exposure to aerosolized pathogens In other cases, however, it is unclear how transmission occurred. Kimman *et al.* notes that ”For the majority of LAIs there appears to be no direct cause” ^52^ - that is, exposures occur even in laboratories that follow standard safety precautions, without obvious accidents.

***Laboratory accidents and known causes*.** A large portion of events involve needle-sticks or other skin punctures. For example, a 2002 event discussed by the NBAF report at an unknown location involving West Nile Virus was an accidental puncture when a researcher was extracting material from a mouse brain. Accidents do not always seem to improve practice, as even after a 2003 anthrax incident involved a University of New Mexico (UNM) researcher, another event, with the pathogen involved redacted, occurred in the same laboratory the following year, 2004 ^20^. Further, not all such accidents are minor - even when an event is known, the infection can be fatal, as in another 2004 event, where Antonina Presnyakova, a researcher at the Russian Vector lab, died after being accidentally stuck by a needle contaminated with Ebola virus ^21^.

Many other diseases are contracted due to insufficient safety procedures around aerosolization. For example, in 2006, at Texas A&M, three students were also infected with Q-fever, presumably due to aerosol challenges done involving livestock. Further safety measures and greater care seem unlikely to fully address this risk. For example, also in 2006, at the same university, a professor at the same university invented a ”foolproof” aerosolization chamber, but during cleaning a researcher contracted Brucella. Compounding the concern, the same chamber was involved in exposures of non-bioweapons pathogens at two other universities the same year ^20^.

Somewhat differently, though still accidental, one laboratory-acquired Dengue event was due to a mosquito bite in a research laboratory in Australia in 2011. This occurred despite wearing proper personal protective equipment, and seemingly involved no mistake on the part of the researcher ^32^.

Safety procedures around facilities and maintenance are another issue that has lead to accidents. In addition to the 1979 Sverdlovsk Anthrax Leak discussed above where a filter was not replaced correctly, a 2007 outbreak of Foot-and-Mouth disease in the UK was with high likelihood traced to aging pipes between two laboratories. In that case, there was a citric acid disinfection procedure in the first laboratory that was known to be insufficient, and the effluent was still categorised as requiring Category 4 containment. Despite this, the pipes leading to the final disinfection stage were not properly inspected or maintained, and a later independent review of the accident found that they very likely leaked ^53^. The result was an outbreak that required the slaughter of herds at four locations, and restricted the export of meat from the UK for several months - with massive economic costs.

It is worth noting that a large number of cases with known details were uncovered due to work by the Sunshine project. This now-defunct project filed requests for non-public records at a large number of universities, only a fraction of which were ever filled. The requested records seem to have covered only the time period between 2003 and the time the records were requested, in 2006. Given that many of these events would presumably have remained unknown without their work, it seems extremely unlikely that the list is complete, and very likely that further such work would expand it.

In a similar vein, the relative paucity of events pre-2003 seems more likely attributable to incomplete records and a lack of public reporting rather than to a lack of events. On the other hand, it seems unlikely that the later time period is representative of the earlier accident rate. This is because there was a significant post-2001 expansion of work related to pathogens that could be used for bioterrorism. While the causes are unclear, it seems plausible both that safety standards were followed less closely in the rush to expand research, and that the relevant class of research was undertaken at a greater pace than earlier, making the same rate of accidents lead to more events.

***Accidents due to unknown causes*.** In some incidents, the cause of infection is less clear, even after later investigation. Janet Parker, the last person to contract Smallpox, died in 1978 due to an escaped pathogen. She was a medical photographer who seemingly never entered the laboratory which worked with Smallpox, but worked a floor above that laboratory. How transmission occurred remains the subject of conjecture.

More recently, in 2009, Dr. Malcolm Casadaban at the University of Chicago died of plague. It is unclear how the infection occurred, but (despite colleague’s claims to the contrary), the University claimed it was due to lax laboratory practices with coats and gloves. Perhaps supporting the claim that the lab did not require sufficient safety precautions, a cutaneous Anthrax infection occurred in the same laboratory in 2011, presumably due to contact with a sample. This was thankfully quickly recognised, perhaps partly due to the earlier death of Casadaban, but it remains unclear when or how exactly the disease was contracted ^21^.

In addition to these cases, there are a number of cases where routine blood testing of laboratory workers uncovered seropositives - indicating that the individual was infected at some point. These infectious are likely subclinical, and (if so) these cases posed minimal risks of spread, but they indicate the existence of many exposure incidents that do not have a known cause, and are only found in retrospect. Given clear reason to believe that not all accidents have known causes, it seems dubious to claim that typical practice is sufficient to eliminate these risks.

***Pathogen mix-ups*.** There have been a number of events where the exposure was due to the accidental presence of a bioweapons pathogen. The Select Agent Program reports also note that there are on the order of one and two hundred events each year where pathogens are lost in transit, misplaced, not properly accounted for in inventories, accidentally destroyed, or otherwise cannot be accounted for, but in general these seem not to have led to exposures or infections. In some cases, however, there were infections or more clear indication of exposure. In 2004, Miller ^54^ reports research on a possible vaccine for anthrax that was supposedly using heat-inactivated Bacillus anthracis. When 49 of the 50 injected mice quickly died, it was realised that the anthrax was not, in fact, inactive, and that the researchers had been exposed - thankfully, none were infected.

In other cases, the pathogen was not supposed to be present at all. In 2005, Johnson ^18^ notes that an event at University of California Berkeley involved accidental exposure to aerosolized Rocky Mountain Spotted Fever instead of the more harmless pathogen they expected. A second similar incident in 2005 involved lab workers at Boston University who were infected with a dangerous variant of Tularemia after working with what they thought was a harmless variant. This seems to be the case referred to in the DHS Select Agent Report, 2003–2006. Prior to the much later revelation of that report due to a FOIA request, this event was publicized by Smith ^10^ due to a scandal involving illegally delayed reporting of Tularemia - and if the reporting had not been delayed, or had never occurred, it seems unlikely that any notice would have been paid, other than (perhaps) the select agent reporting.

Lastly, there have been a number of transit accidents, where a pathogen exposure occurs in transit, or due to a mix-up. For example, there was a dry-ice explosion involving a sample of West Nile Virus being sent by a research group in Columbus, Ohio. Reporting by 30 noted that as many as 50 Federal Express workers may have been exposed.

### Likely missing data and reporting trends

The reports used for sourcing the events in the above list are clearly incomplete, especially for older and undetected events, and international events. There is (unfortunately non-public) reporting of events in the United States, and while it is possible that the greater amount of research in the US means events elsewhere are rarer, it seem hard to believe that international safety standards which for many countries have lagged those in the US - have ensured that no such events occurred.

Evidence that there is under-reporting is scant, for obvious reasons. Despite this, an anonymous survey on biosecurity and accidents was conducted in Belgium provides clear reason to assume such events are common. The survey covered the five-year period from 2007–2012, but these events were not detailed enough for inclusion in the above list. The study uncovered many previously-unknown events, and extrapolated an overall yearly rate of approximately 1,000 bio-accident “possible events,” to adapt the DHS terminology. The survey covered all pathogens, across both research and clinical laboratories, rather than only research, but focused on high risk pathogens. To assist comparison to the above list, only a quarter of respondents worked in BSL-3 laboratories (there are no BSL-4 laboratories in Belgium), and just over half were involved in research and development, rather than clinical work.

One strong piece of evidence of under-reporting is that none of the three previously publicly reported LAIs in Belgium were of Class-3 pathogens, but of the ”74 to 95” additional LAIs reported in Wytsmanstraat’s (anonymous) survey, 19 to 26 involved Class-3 pathogens, including Tuberculosis, Brucella, and Rabies ^55^. These results point to both the obvious under-reporting discussed previously, but also the relatively high prevalence of dangerous pathogen infections.

This leaves the (plausible) possibility that reports of such events were publicly reported but not found in this review of (primarily English-language) reports, or that these incidents are not being publicly reported. On the other hand, there has been work done since the late-aughts to improve biosafety standards.

The Select Agent reports that have been made available via FOIA requests still show a very worrying continuation of 1–2 infection events per year. Reports since 2008 show hundreds of “possible release” events per year, where an exposure may have occurred. This is very relevant for risk analysis involving near-misses and understanding of lab safety. On the other hand, improved reporting should not be taken to imply a greater rate of such events. In fact, the opposite is likely, and better reporting of non-exposure accidents implies that while the number of reported possible events has increased, the number of events has stayed fairly steady or dropped. In recent data, only approximately 1% of reported possible events lead to infections, and a significant and growing portion of these events were sero-conversions with no clinical manifestation, and would have remained unknown without testing - supporting the hypothesized decrease in events.

### Future risks

Not all of the relevant risks are captured by reviewing events. In addition to accidents risks for new types of research recently highlighted by 19, and the info-hazard risks discussed by 56, there are the more general temptation hazard discussed in 57, including the plausible eventuality where something dangerous is done “against our better judgment”, or the even broader risks imposed on humanity from self-interested actors that might rationally choose to deploy biological weapons despite the risks.

The available information about events point to a clear but small risk from laboratory accidents, and a hard-to-quantify risk from both the development of biological weapons, and from research into these pathogens. Even productive basic research, which is demonstrably valuable in mitigating risks, has unfortunately too little attention paid to the risks, which can be significant ^19^.

Given the occasional failure of precautions and safety standards than already exist, the risk posed by the research process itself may in certain cases even outweigh the benefits. Beyond extrapolations from these known events, Howard *et al.* ^58^ argues that it is clear that future directions in laboratory research, including synthetic biology, will pose additional poorly-understood risks of the types reviewed. Even in the present, however, many argue that the risks of research involving gain-of-function for easily spread pathogens is likely to outweigh the benefits, as 59– 61 and others recently suggested regarding gain-of-function work for novel influenza viruses.

***Possible consequences - comparing to influenza accidents*.** None of the historical events listed in the paper involving the highest-risk pathogens created an uncontrolled spread, though one bio-warfare research accident killed more than a hundred people. The same cannot be said for research into influenza, as the 1977–78 Influenza accident showed.

As noted above, Rozo and Gronvall ^23^ detail an event in 1977 that accidentally released the strain of H1N1 influenza that circulated in 1950, and this spread widely. It is still somewhat unclear where or how this event started, but since much of the population had latent immunity due to strains of H1N1 that circulated in 1943 and 1947, the release caused minimal impact on those older than 20, and was thankfully limited.

A worryingly similar event in 2004–5 was reported in 18, 62 and involved the distribution of thousands of what should have been a harmless strain of influenza to labs across the world. This was done as part of testing labs’ ability to identify strains of influenza. Unfortunately, the private company that made the kits included the 1957 Asian influenza instead of a harmless strain. It is plausible that if this strain had escaped, it could have had much more serious consequences there had not been a recently circulating strain of H2N2, and presumably most under the age of 50 were vulnerable. Simulations by Merler et.al. have shown that this risk is non-negligible ^63^, and based partly on this, the report by Klotz in the Bulletin of the Atomic Scientists speculated that “a release into the community of [an avian flu like] pathogen could seed a pandemic with a probability of perhaps 15 percent ^19^.

## Conclusions

The report presents three classes of program, offensive, defensive, and academic research. Each poses risks, and examples of each are available in the recent past.

While this dataset of biological exposure events is nearly certainly incomplete, it is a step towards the needed understanding for assessing the risks of larger scale events. Potentially more importantly, it contributes to building a lower bound for this risk, and points to how much uncertainty remains due to both lax reporting standards and unknowable events.

## Data availability

Table 1 contains the underlying data.

## Footnotes

^1^This research included no further efforts to uncover previously unknown events, though this is likely a useful approach to better understanding the risk.

^2^There were several aborted fetuses due to these infections, but no other deaths.

^3^The incidents of human testing and purposeful use are included in the paper, but given the sensitive nature of these alleged war crimes and/or crimes against humanity, and the inability for those alleging the events to provide inarguable proof, no position is taken on what actually occurred.

^4^A pair of non-attempted “attacks” involving a former white nationalist microbiologist occurred in 1995 and 1998, but the attacker was interested in boasting about his importance rather than planning an attack, and there seems to have been no possibility of any infections at any point ^44^.

^5^As noted earlier, clinical accidents are even more common, but are not the consequence of decisions made about what research to engage in, and the class of accident is very different.

^6^These requests will uncover research accidents, but are not granted for information ”properly classified in the interest of national security,” which could include any events related to (even defensive) biowarfare.
