# Supplementary material for: High-risk human-caused pathogen exposure events from 1975-2016
Source: F1000Res. 2022 Jul 8;10:752. Originally published 2021 Aug 4. [Version 2] doi: 10.12688/f1000research.55114.2 (PMC9274012; doi:10.12688/f1000research.55114.2)
Supplement: Peer review history

# Peer review: Reviewer response for version 2

**DOI:** 10.5256/f1000research.133861.r143686

**Published:** 2022-07-11

**Authors:** Gamal Wareth

**Affiliations:** 1 Institute of Bacterial Infections and Zoonoses, Friedrich-Loeffler-Institut, Greifswald, Germany

I saw the author's responses and I agree and I have no further comments.

Are sufficient details of methods and materials provided to allow replication by others?

Yes

Is the rationale for creating the dataset(s) clearly described?

Yes

Are the datasets clearly presented in a useable and accessible format?

Partly

Are the protocols appropriate and is the work technically sound?

Yes

Reviewer Expertise:

NA

I confirm that I have read this submission and believe that I have an appropriate level of expertise to confirm that it is of an acceptable scientific standard.

# Peer review: Reviewer response for version 1

**DOI:** 10.5256/f1000research.58660.r121971

**Published:** 2022-02-22

**Authors:** Gamal Wareth

**Affiliations:** 1 Institute of Bacterial Infections and Zoonoses, Friedrich-Loeffler-Institut, Greifswald, Germany

The article of Manheim D and Lewis G titled “High-risk human-caused pathogen exposure events from 1975-2016” is interesting and scientifically accepted. The report included a dataset of 71 incidents involving either accidental or purposeful exposure to, or infection by, a highly infectious pathogenic agent. The article is very well written and I recommend indexing it. However, there are minor enquires that need to be addressed before considering indexing.

- The information in table 1 is arranged chronically from the past to the recent while two events (cholera and Anthrax in the 1970s) come later after 1979 and not clear if they have to be listed first in the table as the exact year of the event is not determined.
- In the discussion, section Accidental release, please remove one “due to”, as it repeated two times in the 7 ^th^ line of the paragraph.
- Highlighting the accidental infection of men from vials containing live vaccine candidates during immunization of animals is missing in the report such as what happened in the case of brucellosis.
- Short hints about the COVID-19 story are required which is not clear whether it is a laboratory accident of wildlife acquired infection. Even though all events were included in the table, I expected to see the events classified according to pathogen category e.g. bacterial, viral, to be more clear for the reader.
- Outlooks and recommendations are missing in the conclusion.

Are sufficient details of methods and materials provided to allow replication by others?

Yes

Is the rationale for creating the dataset(s) clearly described?

Yes

Are the datasets clearly presented in a useable and accessible format?

Partly

Are the protocols appropriate and is the work technically sound?

Yes

Reviewer Expertise:

Bioterrorism Agents/Diseases

I confirm that I have read this submission and believe that I have an appropriate level of expertise to confirm that it is of an acceptable scientific standard, however I have significant reservations, as outlined above.

# Response

**Published:** 2022-05-08

**Authors:** David Manheim

**Affiliations:** University of Haifa, Israel

Thank you for your helpful suggestions! In the forthcoming revision, I have added notes addressing  the relevance due to COVID-19, and regarding brucellosis from live vaccines. The absence of outlooks and recommendations in the conclusions is due to the requirements for the journal article type, "data note," intended to describe a dataset rather than make substantive conclusions. If we were called upon to do so, I would note that first, the risks from accidental and deliberate events are very worrying, and require more international cooperation and coordination, and second, the trend towards greater safety is encouraging, but there is significant work remaining for both transparency around accidents and substantive laboratory safety measures which can be decided on the basis of better data, and we hope improvements in laboratory monitoring and safety will continue.

 Regarding chronology, while the dates of some events in the 1970s are unclear, it seems reasonable to put the events which are likely to have occurred or continued later than most of the events during the 1970s at the end of that decade rather than the beginning. (The war in Rhodesia ended in late 1979, while the Sverdlovsk Anthrax event was in April 1979.)

 Finally, thank you for the suggestion to add pathogen types to the data, which I agree can be helpful for those using the dataset.

 My current professional work involves advocacy related to laboratory safety and biorisk policy, and is therefore related to the topic of the research.

# Peer review: Reviewer response for version 1

**DOI:** 10.5256/f1000research.58660.r94609

**Published:** 2021-10-18

**Authors:** Sam Weiss Evans

**Affiliations:** 1 Program on Science, Technology and Society, John F Kennedy School of Government, Harvard University, Cambridge, MA, USA

This article is a Data Note that describes a dataset of 71 incidents involving either accidental or purposeful exposure to, or infection by, a highly infectious pathogenic agent. Building a dataset like this is necessarily non-exhaustive, sporadic, and time-consuming. This paper did not seek to uncover previously unknown events, just gather those that are so far known.

- Is the rationale for creating the dataset(s) clearly described?
- **Response:** Yes. It helps provide evidence for a lower bound on the levels of accidents or purposeful exposure to highly infectious pathogenic agents.
- Are the protocols appropriate and is the work technically sound?
- **Response: **It is. There are numerous reasons why a dataset like this is necessarily incomplete, which are articulated clearly in the paper.
- Are sufficient details of methods and materials provided to allow replication by others?
- **Response: **Yes.
- Are the datasets clearly presented in a useable and accessible format?
- **Response:** Yes, though having an ability to access the table in a more database-friendly manner (e.g. CSV file) would be helpful. 

**Specific comments:**

- p.3 “there is a likely far larger risk that emerges from various intentional human uses of pathogens, especially pathogens that have bioweapon or pandemic potential”: This claim is not supported by the in-text citation, which explicitly notes its comments on anthropogenic extinction causes is highly speculative.
- Inclusion criteria: 
  - “what researchers have referred to as human-caused Global Catastrophic Biological Risks (GCBRs).” Which researchers? Provide citation.
  - “the event involved a pathogen (either wild-type or a enhanced or weaponized variant) that is usable as a bioweapon.” Surely this does not mean that the pathogen was fully weaponized? Please clarify. You could just combine with the following sentence.
  - Regarding South Africa: this would be an opportune spot to mention how, even with South Africa’s inclusion in the BTWC, there was still significant absences with their disclosure of their bio weapons program. See, e.g. Rappert & Gould (2016) “Dis-eases of secrecy.” Jacana Media.
- There should be a different numbering system for footnotes vs endnotes.

Are sufficient details of methods and materials provided to allow replication by others?

Yes

Is the rationale for creating the dataset(s) clearly described?

Yes

Are the datasets clearly presented in a useable and accessible format?

Yes

Are the protocols appropriate and is the work technically sound?

Yes

Reviewer Expertise:

As relevant to this article, I study the processes through which biosecurity governance mechanisms come into being and are seen as legitimate, how they create zones of vision and absence, and how those mechanisms change over time.

I confirm that I have read this submission and believe that I have an appropriate level of expertise to confirm that it is of an acceptable scientific standard.
